# Distinct epidemiologic patterns of mesothelioma: evidence from a 16-year provincial cohort in China

**DOI:** 10.1186/s12885-026-16357-9

**Published:** 2026-06-15

**Authors:** Shiyuan Yin, Tong Hu, Xiao Li, Duo Xu, Yongqian Shu

**Affiliations:** 1 Department of Oncology, The First Affiliated Hospital of Nanjing Medical University, Nanjing, China; 2 Department of Pathology, The First Affiliated Hospital of Nanjing Medical University, Nanjing, China

**Keywords:** Mesothelioma, Cancer epidemiology, Sex distribution, Survival outcomes, Provincial cohort

## Abstract

**Background:**

Epidemiologic data on mesothelioma in China remain limited, particularly with respect to long-term, province-level characterization of demographic features and survival outcomes.

**Methods:**

We conducted a 16-year retrospective cohort study of mesothelioma patients diagnosed at a major provincial tertiary referral center in China between 2009 and 2025. Among 128 identified cases, 93 met strict pathological criteria and were included for analysis. We evaluated sex distribution, anatomic site, age at diagnosis, and survival outcomes, and compared these features with representative cohorts from the United States, Europe, and Japan.

**Results:**

Our 16-year provincial cohort showed a nearly equal male-to-female ratio that markedly distinct from the male predominance reported in Western populations. Peritoneal mesothelioma accounted for 33.3% of cases, a proportion higher than that typically reported in global cohorts. The higher proportion of female patients was observed across both pleural and peritoneal mesothelioma. The median age at diagnosis was significantly younger than that observed in the United States. The median progression-free survival (PFS) and overall survival (OS) were 10 months and 16 months, with a notable survival advantage for females with peritoneal mesothelioma. Notably, only 7.5% of patients had documented asbestos exposure, indicating additional environmental and/or genetic contributors maybe relevant in this setting.

**Conclusions:**

This long-term provincial cohort delineates mesothelioma epidemiology at the province level in China, marked by a near-equal sex distribution with substantial female representation across disease subtypes, a high proportion of peritoneal cases, younger age at diagnosis and a low prevalence of documented asbestos exposure. These findings show clear region-specific evidence, underscoring potential geographic heterogeneity in mesothelioma epidemiology.

**Supplementary Information:**

The online version contains supplementary material available at 10.1186/s12885-026-16357-9.

## Introduction

Mesothelioma is a rare yet highly aggressive malignancy. By anatomic site, it is mainly classified into pleural, peritoneal, and pericardial mesothelioma [1]. To date, two major etiological factors are well established: exposure to asbestos through mining or environmental contact, and inherited susceptibility due to germline variants in *BAP1* and *BRCA2* [2, 3]. Growing evidence indicates that carriers of pathogenic variants in *BAP1*, *PALB2*, *BRCA1*, *FANCI*, and *ATM* exhibit increased susceptibility to pleural mesothelioma with asbestos exposure [4], supporting a gene-environment interaction. Because talc and asbestos may co-occur in natural deposits, fibrous talc is regulated as asbestos by Occupational Safety and Health Administration (OSHA) and classified as carcinogenic by International Agency for Research on Cancer (IARC) in the United States [5]. Additional putative risk factors, including erionite exposure and prior chest radiotherapy, have also been linked to the development of mesothelioma [1, 6, 7].

Against this etiologic backdrop, China remains one of the few large countries with ongoing asbestos use [8]. Large chrysotile mines are found in Sichuan, Liaoning, Qinghai, and Hebei, while asbestos fiber processing industries are clustered in Anhui, Hebei, and Zhejiang [9]. Although national mesothelioma surveillance is being refined [10], documented asbestos exposure is uncommon in routine records, and detailed provincial-level analyses focusing on sex distribution, mesothelioma subtypes and survival indicators remain limited.

To address this gap, we analyzed a 16-year cohort of mesothelioma cases treated at Jiangsu Province Hospital, a major provincial tertiary referral center. We systematically characterized sex distribution, anatomic site, age at diagnosis, survival outcomes, and documented asbestos exposure, and compared these metrics with representative cohorts from the United States, Europe, and Japan [11–14]. By providing long-term, province-level data from a highly populated region of China, this study characterizes demographic and clinicopathological patterns of mesothelioma that have been insufficiently described to date.

## Methods

### Study population and inclusion criteria

This retrospective study included patients diagnosed with mesothelioma between January 2009 and December 2025. Eligible patients met the following inclusion criteria: (1) histopathologically confirmed mesothelioma; (2) age between 18 and 85 years at the time of diagnosis; (3) complete clinical and treatment records; and (4) availability of disease evaluation according to the modified Response Evaluation Criteria in Solid Tumors (modified RECIST) for pleural mesothelioma [15]. Patients without definitive pathological confirmation or with substantial missing clinical or treatment data were excluded from the study.

### Data sources and geographic distribution

We analyzed the incidence of mesothelioma in eastern China from 2009 to 2025, with data primarily sourced from Jiangsu Province Hospital. Approximately 80% of the cases resided in Jiangsu, with other cases came from Anhui, Shanghai, Shandong and other provinces. Clinical variables, including sex, age, disease stage, treatment information, and survival time, were extracted from medical records and included in all demographic and survival analyses. Survival time was calculated from the date of pathological diagnosis. Radiological features were derived from CT and/or PET-CT scans obtained at initial presentation and were systematically evaluated by experienced radiologists, with particular emphasis on characteristics like pleural plaques, pleural nodules or masses, pleural thickening and pulmonary fibrous streaks. Verbal informed consent was obtained from all patients. Both male and female patients were eligible for inclusion, and sex was considered as a biological variable in subsequent demographic and survival analyses.

### Diagnosis

One hundred twenty eight cases diagnosed as mesothelioma (ICD10:C45) were collected and 93 cases were confirmed by pathology. The ratio of percutaneous biopsy, surgical biopsy (including endoscopic sampling), and liquid-based cytology for diagnosis is approximately 6:7:1. The pathological diagnosis is established based on hematoxylin and eosin (HE) staining and immunohistochemical staining results, including markers such as Calretinin, CK5/6, WT1, and D2-40 [16]. The pathological diagnoses were all determined by experienced pathologists. A total of 93 patients with pathologically confirmed diagnoses were enrolled in the final statistical analysis. Date of diagnosis is defined as the date of the pathology report. Histological subtype information was extracted from pathology reports when available and included in the corresponding analyses.

### Genomic profiling and variant interpretation

Tumor genomic profiling was performed using multiple specimen types, including formalin-fixed, paraffin-embedded (FFPE) tumor tissues and cell blocks derived from pleural and ascitic effusions. Most cases were analyzed using a targeted sequencing panel covering 196 cancer-related genes, while a subset of patients (2/9) underwent sequencing with expanded panels encompassing 520 or 566 genes. Matched normal control samples were available for only one patient, in whom oral mucosal tissue was collected to enable germline mutation analysis. In all other cases, detected variants were interpreted as somatic.

Somatic variants were classified into Tiers I–IV according to the joint consensus recommendations of the Association for Molecular Pathology, American Society of Clinical Oncology, and College of American Pathologists. Briefly, Tier I variants were defined as having strong clinical significance; Tier II, potential clinical significance; Tier III, uncertain clinical significance; and Tier IV, benign or likely benign. Germline variants were classified as pathogenic, likely pathogenic, variants of uncertain significance, likely benign, or benign according to the guidelines of the American College of Medical Genetics and Genomics in conjunction with the Association for Molecular Pathology (2015).

### Data analysis

All statistical analyses and data visualizations were performed using R software (version 4.3.2). Categorical variables, including sex distribution and anatomical subtype, were compared using the chi-square test for independence, assuming mutually exclusive categories and independent observations. Age differences between groups were evaluated using unpaired, two-sided independent-samples t-tests after assessment of approximate normality. Overall survival was estimated using the Kaplan–Meier method, and differences between groups were compared using the log-rank test, assuming independent survival times and non-informative censoring. A locally estimated scatterplot smoothing (LOESS) method was applied to model temporal trends in annual case counts. No a priori power calculation was performed, as this was a retrospective, observational study including all eligible patients diagnosed during the study period. All statistical tests were two-sided, and a P value < 0.05 was considered statistically significant.

## Results

### Sex, anatomical distribution and histological type of mesothelioma

**Fig. 1:**
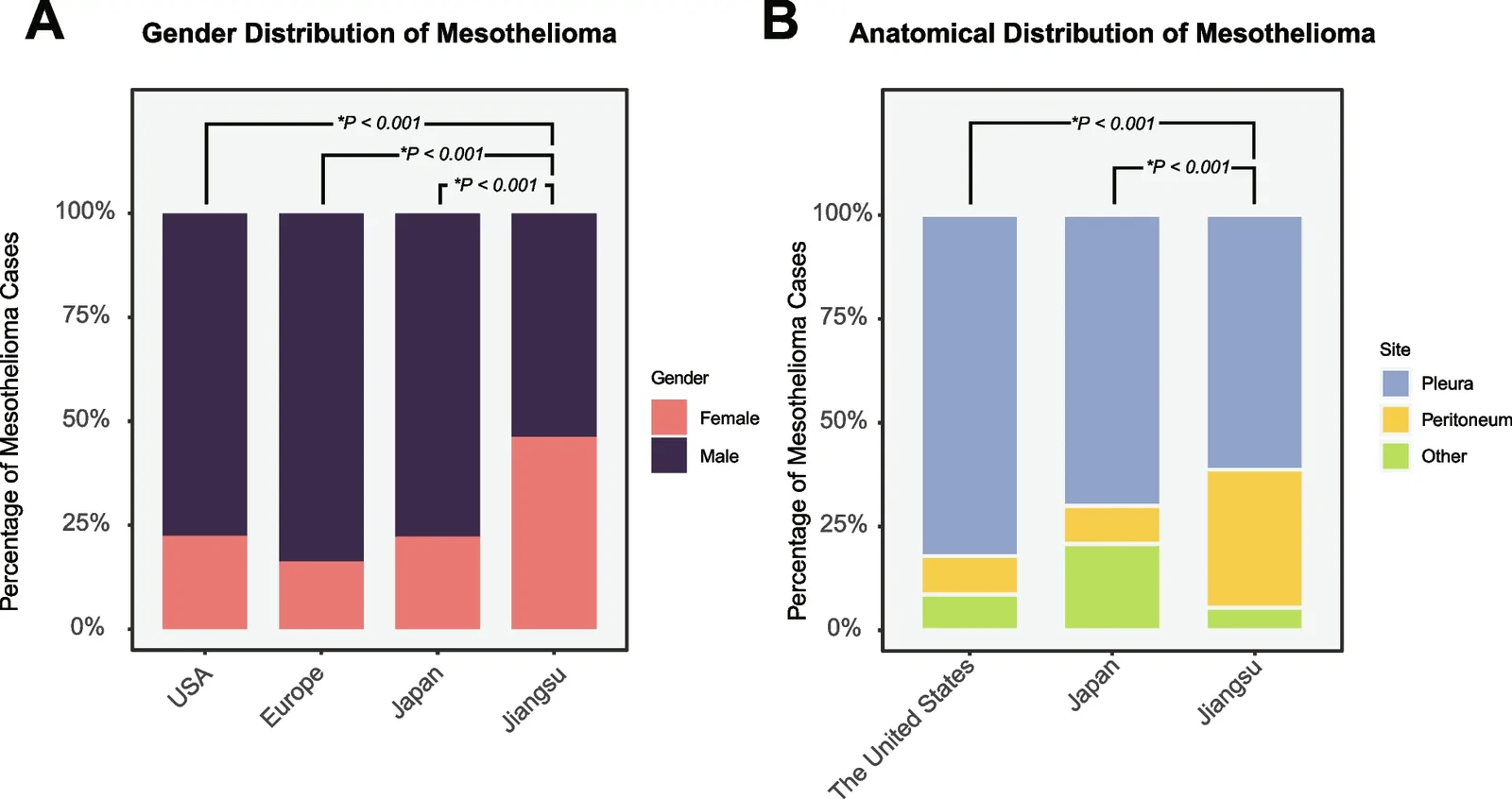
Gender and anatomical distributions of mesothelioma in Jiangsu Province compared with other regions. **A** The gender distribution patterns in mesothelioma were compared between Jiangsu and the United States, Europe, and Japan. The proportion of female patients in Jiangsu was significantly higher than in the each of these regions. (The United States: χ^2^ = 28.994, *P* < 0.001; Europe: χ^2^ = 57.969, *P* < 0.001; Japan: χ^2^ = 28.615, *P* < 0.001). **B** Statistically significant differences were found in the anatomical distribution patterns of mesothelioma when comparing Jiangsu Province with the United States and Japan (the United States: χ^2^ = 886.296, *P* < 0.001; Japan: χ^2^ = 87.209, *P* < 0.001). Differences were assessed by χ.^2^ test (two-sided *P* < 0.05 considered significant)

We identified 93 pathologically confirmed mesothelioma cases (50 males and 43 females), yielding an approximate 1:1 male-to-female ratio. By contrast, sex ratios reported in the United States (3.48:1), Europe (5.17:1), and Japan (3.51:1) are markedly higher (Fig. 1A). These findings indicate a substantially lower male predominance in our cohort compared with Western and Japanese reports.

Among the 93 cases, the distribution of mesothelioma types was as follows: pleural mesotheliomas constituted 61.29%, peritoneal mesotheliomas 33.33%, and mesothelioma arising from other anatomical sites for the remaining 5.38%. Notably, the pleura-to-peritoneum ratio in our cohort was ~ 1.83:1, which is far lower than that reported for the United States (8.90:1) and Japan (7.71:1) (Fig. 1B). Though European data were excluded due to unavailability, statistics from both the Netherlands and Norway indicate an overwhelming male predominance (the Netherlands: 18.34:1; Norway: 16.54:1) [17, 18]. While our cohort still showed a predominance of pleural mesothelioma, consistent with these international patterns, the higher proportion of peritoneal cases underscores potential regional differences within China.

When stratified by mesothelioma subtype, the overall sex distribution did not differ significantly (*P* = 0.40) (Figure S1A). Within the pleural mesothelioma subgroup, the male-to-female ratio in our cohort (57.89% vs 42.11%) was lower than that reported in the U.S. cohort (80.1% vs 19.9%), indicating a less pronounced male predominance (Figure S1B). In peritoneal mesothelioma, the sex distribution in Jiangsu (48.39% vs 51.61%) was broadly comparable to that observed in the United States (57.5% vs 42.5%) (Figure S1C).

Histological subtype information was available for 36 of 93 patients and was included in the following analysis. Among these patients, epithelioid mesothelioma was the predominant subtype (23/36, 63.89%), followed by sarcomatoid mesothelioma (12/36, 33.34%), while biphasic mesothelioma was rare (1/36, 2.78%). No significant sex-based differences in histological subtype distribution were observed (*P* = 0.42) **(**Figure S1D).

### Age at diagnosis

**Fig. 2:**
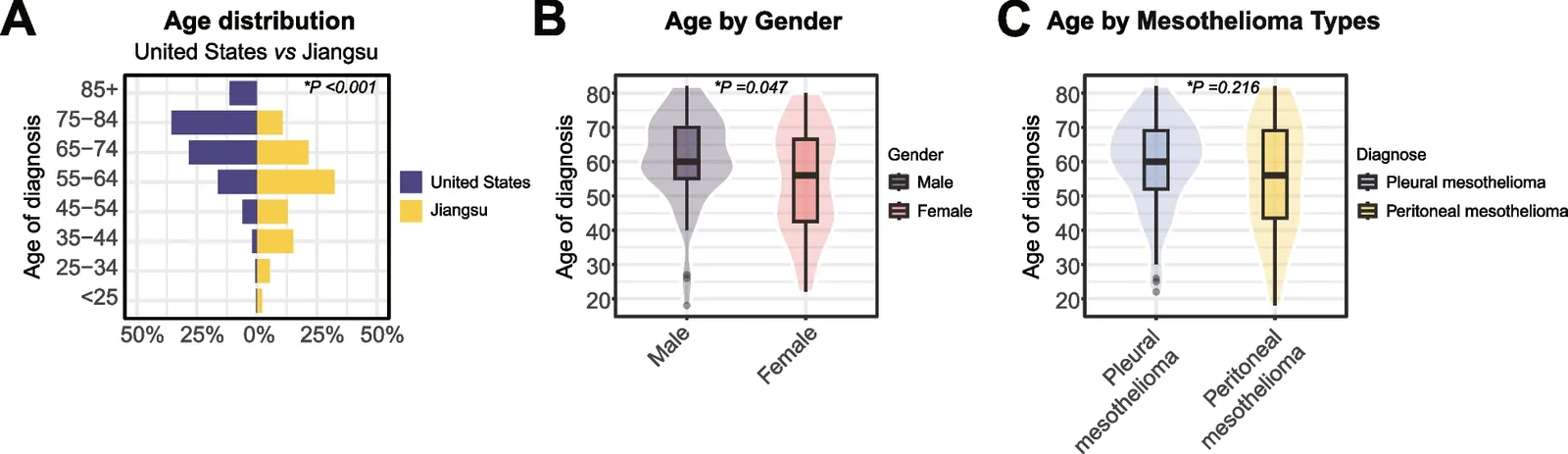
Age distribution by region, gender and mesothelioma subtype. **A** The age distribution pyramids of mesothelioma patients in Jiangsu (56.67 years, SD: 14.93, 95% CI: 53.59–59.74) and the United States (71.7 years, SD: 12.3, 95%CI:77.97–78.35), compared by a χ^2^ test, show a significant difference (χ.^2^ = 172.98, *P* < 0.001). **B** Comparison of age distribution between males and females in Jiangsu, showing a statistically significant difference (Male: 53.4 years, SD: 15.6, 95% CI: 48.6–58.2; Females: 59.5 years, SD: 13.9, 95% CI: 55.5–63.5; t = −2.006, *P* = 0.047). **C** No significant difference in age distribution between pleural and peritoneal mesothelioma groups (PM: 58.4 years, SD: 13.8, 95% CI: 54.7–62.0; PEM:54.2 years, SD: 17.0, 95% CI: 48.0–60.4; t = −1.247, *P* = 0.216). Age differences between patients were analyzed using an independent samples t-test after testing for homogeneity of gender and mesothelioma type variances. Statistical significance was defined as two-sided *P* < 0.05

The median age at diagnosis in our cohort was determined to be 56.67 years (95% CI: 53.59–59.74), which is significantly lower than the median age reported in the United States, which stands at 71.7 years (SD: 12.3; χ^2^ = 172.98,95%CI:77.97–78.35, *P* < 0.001) (Fig. 2A). Due to the lack of age-specific data for Europe and Japan, they were not included in the comparison.

Additionally, a significant difference in the age at diagnosis was noted between genders within our cohort. The mean age at diagnosis for females was 53.4 years (95% CI: 48.6–58.2), which is lower than that for males, who had a mean age of 59.5 years (95% CI: 55.5–63.5, *P* = 0.047) (Fig. 2B). Moreover, no significant differences in age at diagnosis were found between cases of pleural mesothelioma (58.4 years, 95% CI: 54.7–62.0) and peritoneal mesothelioma (54.2 years, *P* = 0.216) (Fig. 2C). In summary, our cohort was diagnosed at a younger age than U.S. series, with women presenting younger than men, and no age difference between pleural and peritoneal disease.

### Survival outcomes

**Fig. 3:**
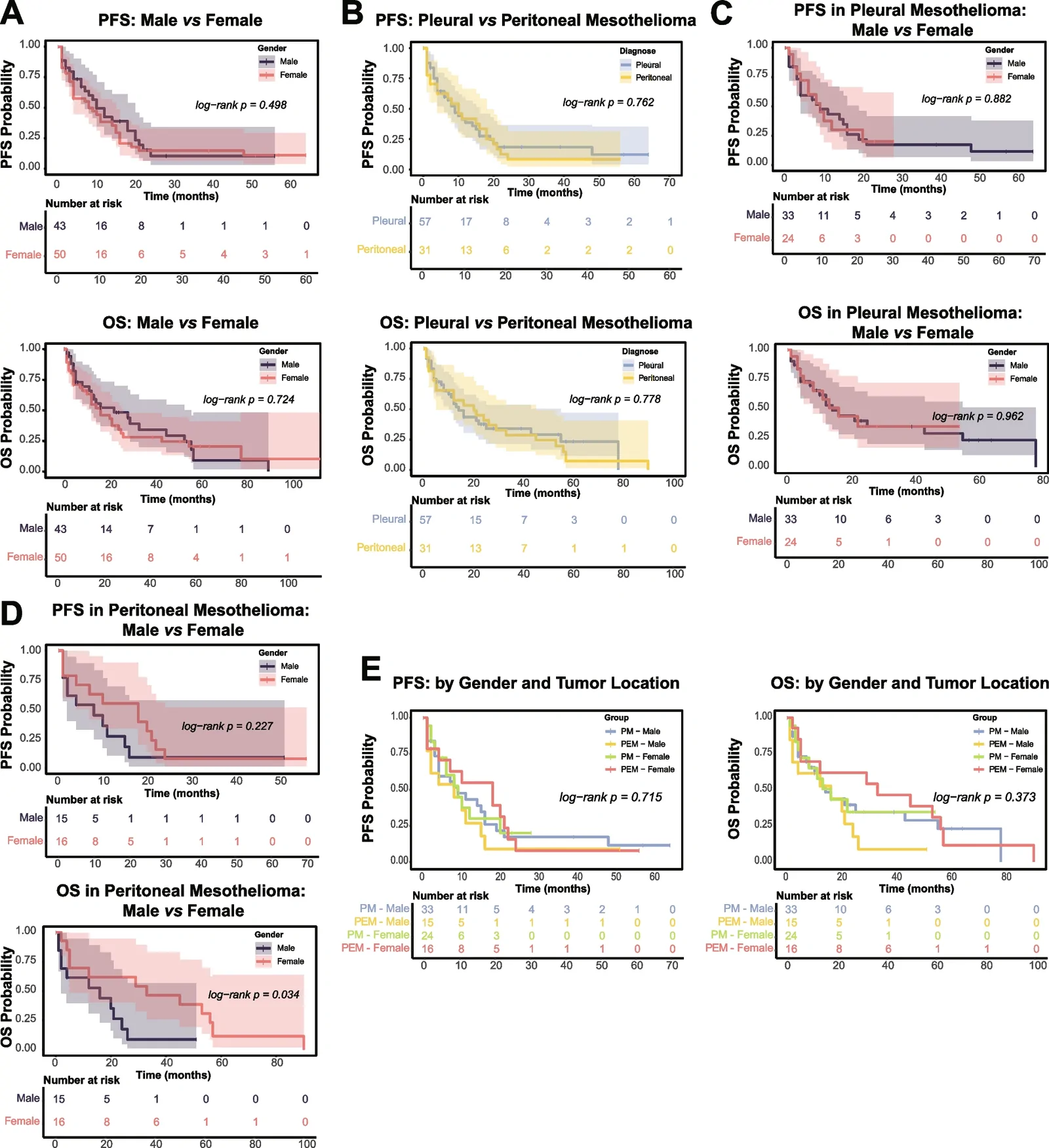
Kaplan–Meier survival curves of progression-free survival (PFS) and overall survival (OS) by gender and mesothelioma subtype. **A** PFS (*P* = 0.498) and OS (*P* = 0.724) by gender. **B** PFS (*P* = 0.762) and OS (*P* = 0.778) by mesothelioma subtype (pleural *vs*. peritoneal). **C** Gender-based differences in PFS and OS within pleural and peritoneal mesothelioma. In peritoneal mesothelioma, a significant difference in OS was observed between male and female patients (*P* = 0.034), whereas PFS did not differ (*P* = 0.227). In pleural mesothelioma, no significant gender differences were observed in either OS (*P* = 0.962) or PFS (*P* = 0.882). **D** PFS (*P* = 0.715) and OS (*P* = 0.373) by both gender and anatomical location. **E** A comprehensive comparison of PFS and OS by gender (male and female) and mesothelioma type (pleural and peritoneal). Survival outcomes were assessed using Kaplan–Meier curves, and differences between groups were evaluated with the log-rank test. PFS: Progression Free Survival, OS: Overall Survival, PM: Pleural Mesothelioma, PEM: Peritoneal Mesothelioma

In our cohort, the median progression-free survival (PFS) was 10 months (95% CI: 7–15), and the median overall survival (OS) was 16 months (95% CI: 12–28). No significant differences in PFS or OS were observed between males and females overall (Fig. 3A), nor between pleural and peritoneal mesothelioma subtypes (Fig. 3B).

When stratified by anatomical subtype, no sex-based survival differences were identified among patients with pleural mesothelioma, no sex-based survival difference was found among pleural mesothelioma patients (Fig. 3C). In contrast, within the peritoneal mesothelioma subgroup, female patients demonstrated a significantly longer OS than male patients (median OS: 33 vs. 16 months; *P* = 0.03) (Fig. 3D), while PFS did not differ significantly.

Further stratification by both sex and tumor location revealed no statistically significant differences in survival among the four subgroups; however, female patients with peritoneal mesothelioma consistently showed the most favorable survival trends, whereas male patients with peritoneal mesothelioma exhibited the poorest outcomes (Fig. 3E).

### Genomic alterations

A subset of patients (17/93) in our cohort underwent targeted genomic profiling, of whom 11 harbored detectable genomic alterations. Among these, eight patients carried clinically significant somatic variants (Tier I–II) involving *TP53, EWSR1, NF2, CDKN2A, BRCA*, and *ROS1*. Additionally, concurrent germline variants in *BAP1* and *BARD1* were identified in one patient. These findings underscore the potential clinical relevance of comprehensive genomic profiling in Chinese patients with mesothelioma (Table S1).

### Asbestos exposure and radiological findings

We further assessed asbestos exposure within the cohort. Based on follow-up data, only 7.5% of patients reported a definite history of asbestos exposure. Given that asbestos-related thoracic malignancies are frequently associated with radiological features such as pleural plaques and pulmonary fibrosis [19], we performed a focused imaging analysis in patients with pleural mesothelioma. Among the 57 patients with pleural mesothelioma, non-calcified pleural nodules were identified in 47.4% (27/57), while pleural calcified plaques were observed in 5.26% (3/57) of cases. Pleural thickening was present in 43.9% (25/57) of patients, and 10.5% (6/57) exhibited pleural masses. Six patients showed no obvious pleural abnormalities or presented solely with pleural effusion at initial presentation. In addition, fibrotic streaks within the lung parenchyma were detected in 47.4% (27/57) of patients (Figure S1E).

### Trends in mesothelioma case volume

**Fig. 4:**
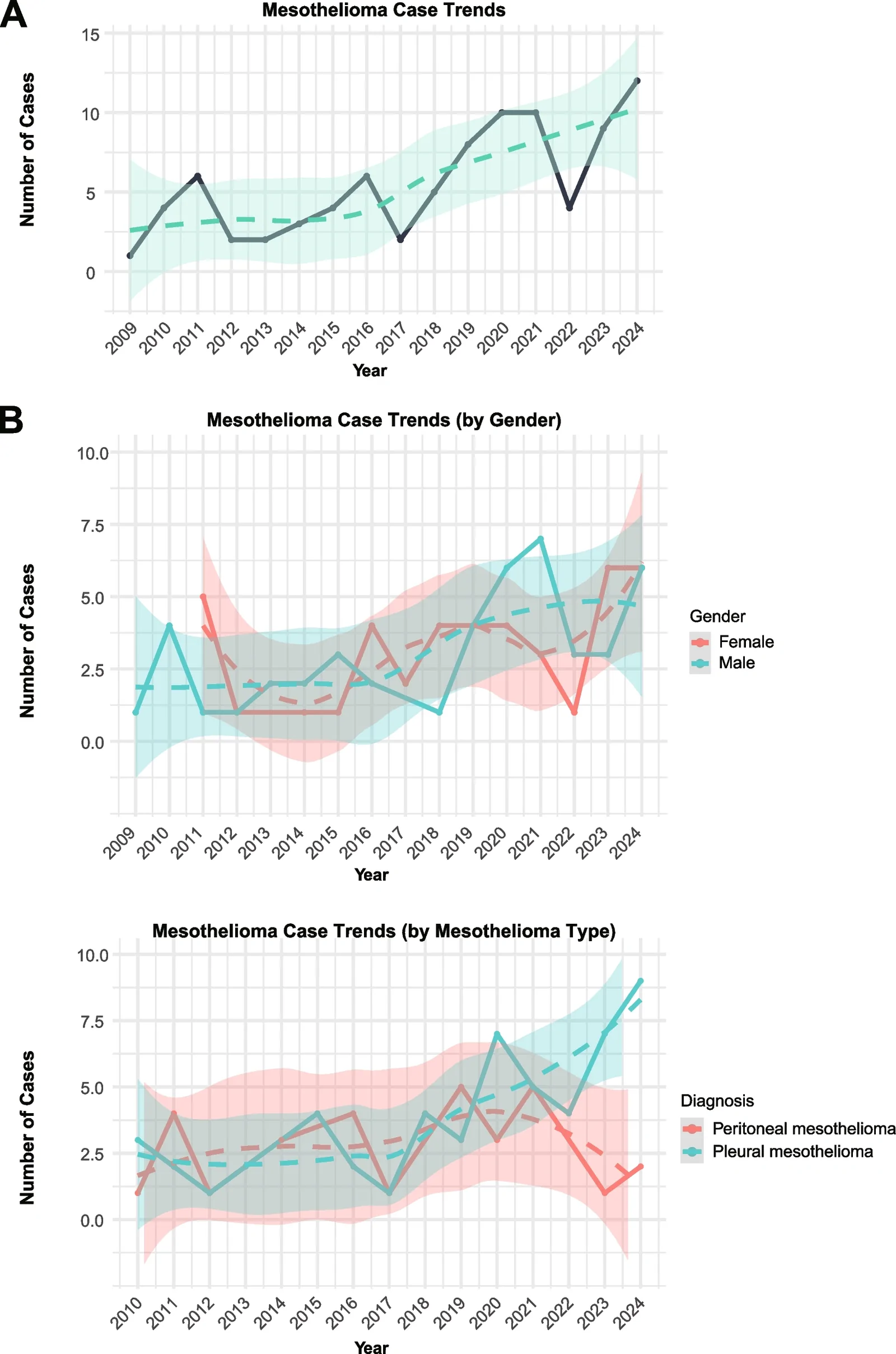
Trends in mesothelioma cases in the Jiangsu cohort from 2009 to 2024. **A** Temporal trends in annual case numbers from 2009 to 2024. **B** Comparative analysis of incidence trends stratified by gender and mesothelioma subtype. Due to incomplete data for 2025, the analysis was restricted to cases diagnosed up to 2024. The shaded area represents the 95% confidence interval

We analyzed the annual number of mesothelioma cases treated at Jiangsu Province Hospital between 2009 and 2024 and assessed temporal trends using curve fitting. Over this period, the total number of treated cases showed an overall increase (Fig. 4A). When stratified by sex, the number of male cases increased during the earlier years of the study and declined in more recent years, whereas no clear temporal trend was observed among female patients. Analysis by anatomic subtype showed different patterns over time: pleural mesothelioma cases increased gradually in recent years, while the number of peritoneal mesothelioma cases remained relatively stable throughout the study period (Fig. 4B).

## Discussion

In this long-term provincial cohort, we identified several epidemiologic characteristics of mesothelioma that differ from patterns reported in international series. The sex ratio was ~ 1:1, contrasting with the male predominance reported in the United States, Europe and Japan. Although pleural mesothelioma remained the predominant anatomic subtype, peritoneal disease accounted for approximately one-third of cases. Female patients were represented across both pleural and peritoneal mesothelioma rather than being confined to a single subtype. Patients in our cohort were younger at diagnosis than in European and U.S. cohorts, and women were diagnosed younger than men. Clinically, median PFS/OS were 10 and 16 months; among peritoneal cases, women lived longer than men (33 vs 16 months; *P* = 0.03), with no sex difference in pleural disease. Over the 16-year study period, overall case numbers increased, primarily driven by pleural mesothelioma, while peritoneal case counts remained relatively stable. Together, these findings delineate region-specific mesothelioma patterns at the provincial level in China.

### Limited asbestos exposure highlights the role of gene-environment interactions in mesothelioma

Globally, the higher incidence of mesothelioma in males is largely attributed to occupational asbestos exposure [20]. However, in China, females constitute a significant proportion of mesothelioma cases. Consistent with this observation, large population-based analyses have reported that a substantial proportion of female mesothelioma patients lacked identifiable occupational asbestos exposure, supporting the contribution of non-occupational and host-related factors [21]. Previous studies have reported that in areas with intensive asbestos mining and processing, nearby residents and farmers can also be exposed to high concentrations of asbestos dust [9].

China is the world’s second-largest producer and consumer of asbestos-based products and holds the third-largest global asbestos reserves [22]. Statistical analysis reveals that 78.4% of asbestos-related enterprises (AREs) located in eastern China, with the majority engaged in processing raw asbestos materials and manufacturing primary asbestos products [22]. In contrast, most asbestos deposits in China are concentrated in northern and northwestern regions [9], whereas no known deposits of asbestos or erionite have been identified in Jiangsu Province, indicating a geographic mismatch between resource distribution and industrial activity. From 2009–2024, case counts at our center rose overall, with male counts increasing and female counts relatively stable, even as national asbestos use has been gradually reduced [23]. This pattern is consistent with legacy and non-occupational exposures, long latency, and under-ascertainment of exposure histories, rather than with occupational exposure alone.

In our cohort, only 7.5% of patients had documented asbestos exposure; most denied occupational or environmental contact. This may partially be attributed to limited public awareness of asbestos-containing materials. As asbestos-related thoracic malignancies often exhibit radiological features such as pleural plaques and pulmonary fibrosis [19], the imaging findings in our cohort raise the possibility that a substantial proportion of patients with pleural mesothelioma may have had prior asbestos exposure. However, due to the limited availability of adequate lung tissue specimens, we were unable to assess ferruginous bodies on histology, another characteristic indicator of asbestos exposure [24].

Other determinants may also be relevant: genetic susceptibility and geogenic fibers. Indeed, 11.8% of patients were farmers, raising the possibility of environmental contact with geogenic asbestos or other carcinogenic fibers [1]. Growing evidence indicates that asbestos can accelerate tumorigenesis on a background of pathogenic variants; thus, gene-environment interaction, rather than either factor alone, is another plausible explanation in this setting. Finally, data on asbestos exposure were based on patient self-report and may be subject to underreporting or recall bias, which may introduce potential bias and should also be taken into consideration.

### The status and needs for genetic testing in mesothelioma in China

Although pleural mesothelioma remains the predominant, the proportion of peritoneal mesothelioma in China is significantly higher compared to the United States and Japan. Emerging evidence suggests a tight link between peritoneal mesothelioma and specific genetic alterations, including loss of *BAP1* protein expression (57%), hemizygous *NF2* loss (35%), and homozygous *CDKN2A* deletions (29%) [25].

Genetic testing is not yet routine in China; coverage in our series was limited and most assays used lung-cancer-oriented rather than mesothelioma-specific panels, constraining interpretation. Thus, implementing dedicated panels and standardized germline pathways is urgently needed to improve prognostic stratification and support cascade testing in families. In this context, the identification of individuals with a genetic predisposition to mesothelioma has important clinical implications [1]. Hereditary cases (e.g., *BAP1*-associated) may follow a less aggressive clinical course and could be more amenable to curative-intent treatment if detected early [26]. Given their elevated risk of multiple malignancies, structured surveillance with periodic imaging and clinical assessment may be warranted, despite the current lack of standardized protocols.

### Regional variations in mesothelioma patterns across China

Notably, our findings indicate regional heterogeneity in mesothelioma within China. Prior provincial-level data from eastern China (2002–2015) reported a markedly different gender distribution and a higher proportion of peritoneal mesothelioma than observed in our Jiangsu cohort [21] (Supplementary Fig. 2), underscoring variability even among geographically proximate regions.

This heterogeneity is further supported by recent multicenter analyses [27], which demonstrated pronounced regional differences in mesothelioma patterns across China in relation to asbestos type, industrial use, and geographic exposure history. In that study, pleural mesothelioma was predominantly associated with crocidolite exposure in mining regions such as Yunnan, whereas peritoneal mesothelioma clustered in areas with hand-spun chrysotile use, particularly in Zhejiang Province. Consistently, a multi-center real-world cohort from major referral centers in northern China (2007–2024) also reported distinctive demographic and clinicopathologic features in Chinese patients, including a higher proportion of females and younger age at diagnosis, low reported asbestos exposure, and a predominance of pleural over peritoneal disease [28].

Notably, Jiangsu was minimally represented in these multicenter datasets, limiting their ability to characterize mesothelioma patterns in this highly populated province. These contrasts highlight the value of province-level, longitudinal data for capturing mesothelioma patterns in regions not adequately represented by existing multicenter studies.

### Differences in mesothelioma between China and other countries

Based on the analysis described above, the major differences in mesothelioma between China and other countries are reflected in three main aspects: a higher incidence rate among female patients, a larger proportion of peritoneal mesothelioma, and a younger age of onset. An intriguing observation is that the median age at diagnosis in this cohort is more than a decade earlier than that reported in the United States. This may suggest a genetic predisposition or environmental exposure since childhood [26, 29]. Further investigation is warranted to elucidate the relative contributions of these factors.

On one hand, genetic factors may be a reason for this disparity, which have been proven to be instrumental in gender disparities [30, 31]. Moreover, studies have revealed that occupational asbestos exposure plays a more limited role in the pathogenesis of peritoneal mesothelioma [32], indicating that other factors, including genetic susceptibility or anatomical differences, may be involved. On the other hand, environmental asbestos exposure is also likely to play an important role. Although most patients in this cohort denied a history of asbestos exposure, historical data indicate widespread use of asbestos-containing materials in rural areas of Jiangsu Province during the 1980s [22, 33]. These materials remained in use until approximately 2010, when demolition and replacement began following updated national standards [33]. Such prolonged environmental exposure may have contributed to asbestos exposure outside traditional occupational settings.

Consistent with this, occupational data available for a subset of female patients (*n* = 20) showed that the majority were not employed in traditionally high-risk occupations. Farmers constituted the largest group (30%), followed by accountants, teachers, white-collar workers, and textile workers (each 15%), while marketing personnel and students accounted for smaller proportions. These findings support the possibility that environmental, rather than occupational, asbestos exposure may contribute to the higher proportion of female mesothelioma cases in this cohort. Evidence from environmentally exposed populations, including studies by Metintas et al., suggests a tendency toward younger age at diagnosis [34, 35]. Such early-life or prolonged environmental exposure may partially explain the younger age of onset observed in eastern China.

In addition, our cohort showed a relatively high proportion of peritoneal mesothelioma cases. The potential role of environmental exposure in the development of peritoneal mesothelioma warrants further investigation. At the same time, increasing evidence points to substantial regional heterogeneity within China, indicating that cross-national comparisons should be interpreted considering local exposure histories.

Overall, these differences likely reflect the combined effects of environmental and biological factors, which together contribute to the distinct epidemiological characteristics of mesothelioma in China compared with other countries.

### Survival disparities in mesothelioma by gender

Our survival analysis in the whole cohort demonstrated median PFS and OS were 10 and 16 months, respectively. Furthermore, we observed a pronounced sex-based disparity in OS, with female patients demonstrating significantly longer survival than male. Notably, a survey from the Washington Cancer Institute revealed that female patients with peritoneal mesothelioma also had a more favorable prognosis than males [36]. We speculate that genetic factors may play a critical role in the observed sex-based survival disparities, particularly after considering multifactorial nature of disease outcomes including tumor stage, age or histologic subtype. Recent study report that germline *BAP1*-associated mesothelioma exhibits distinctive histology and demonstrates markedly lower aggressiveness compared to asbestos-induced mesothelioma [37, 38]. Moreover, genetic alterations in *BAP1*, *NF2* and *CDKN2A* have been shown to significantly impact survival [25, 39]. These observations suggest that sex-based distributions of genetic mutations may partly explain the survival difference. Another investigation elucidated that for mesothelioma, comprehensive molecular analysis is highly valuable in providing patients with personalized treatment recommendations [40]. Further studies with larger sample sizes and comprehensive genomic profiling are warranted to clarify the mechanisms underlying this disparity.

Regarding treatment, most patients in our cohort were managed with upfront systemic therapy without surgical intervention (Table 1). Among patients who received chemotherapy, 40.0% received anti-angiogenic combination, and 32.3% received chemotherapy in combination with immunotherapy. Moreover, it should be noted that treatment strategies varied across patients and were not standardized, which may have influenced survival outcomes.

**Table 1:** Summary of the number and percentage of patients receiving different therapeutic modalities and the agents involved

(A) Number and percentage of patients with different therapies			
**Therapy**		**Number**	**Percentage**
S (only)		4	4.30%
S + C		8	8.60%
S + C + A		1	1.08%
S + C + A + I		3	3.23%
C (only)		32	34.40%
C + A		12	12.90%
C + I		7	7.53%
C + A + I		14	15.05%
A (only)		1	1.08%
I (only)		2	2.15%
NO treatment		6	6.45%

**(B) Therapeutic agents involved**			
**Therapy**	**Medicine**		
Chemotherapy	pemetrexed combined with platinum-based agents, gemcitabine, epirubicin, paclitaxel		
Antiangiogenic therapy	bevacizumab or anlotinib		
Immunotherapy	sintilimab, cadonilimab, camrelizumab, pembrolizumab, ipilimumab		

*S* Surgery, *C* Chemotherapy, *A* Antiangiogenic therapy, *I* Immunotherapy

(A) Distribution of patients according to therapeutic modality, including surgery, chemotherapy, antiangiogenic therapy, and immunotherapy. This classification represents the cumulative number of treatment types administered to each patient over the entire course of therapy

(B) Primary therapeutic agents used within each therapeutic category

Due to the lack of detailed questionnaire-based assessments, patients in this study may have had difficulty accurately recalling or identifying prior asbestos exposure, potentially leading to an underestimation of its prevalence. Furthermore, this cohort was based on previously established diagnoses of mesothelioma. Given that misdiagnosis has been reported in eastern China, particularly for pleural mesothelioma [41], the present cohort may not fully represent the complete disease spectrum. Therefore, these findings should be interpreted with caution.

## Conclusion

Using long-term, province-level data, this study defines a distinct epidemiologic profile of mesothelioma in China that differs from international series, highlighting substantial geographic heterogeneity. These findings provide a stable provincial reference and support the value of region-, sex-, and site-specific approaches in mesothelioma surveillance and comparative epidemiologic research.

## Supplementary Information

Supplementary Material 1. Supplementary Figure 1. Combined analysis of gender and mesothelioma subtypes. (A) The overall distribution of mesothelioma subtypes was similar between males and females, with no significant difference (χ² = 2.943, *P* = 0.40). (B, C) Regional variations in gender-specific proportions of pleural and peritoneal mesothelioma. A significant difference in gender distribution was observed between Jiangsu and the United States in the pleural mesothelioma group (χ² = 16.172, *P* < 0.001). (D) Distribution of histological subtypes of mesothelioma stratified by sex, showing no significant difference between male and female patients (χ² = 1.7246, *P* = 0.42). (E) Proportional distribution of radiological findings identified in pleural mesothelioma. “Pleural non-specific” is defined as the absence of identifiable pleural abnormalities or the presence of isolated pleural effusion at initial presentation. Differences were analyzed using the χ² test, with two-sided *P* < 0.05 considered statistically significant.

Supplementary Material 2. Supplementary Figure 2. Comparison between Jiangsu and Zhejiang provinces in China. (A, B) The gender and anatomical distribution patterns in mesothelioma were compared between Zhejiang and Jiangsu provinces in China, with both comparisons reaching statistical significance (*P*<0.001). (Gender: χ² = 15.004, *P*<0.001; Anatomical distribution: χ² = 25.972, *P*<0.001). Differences were analyzed using the χ² test, with two-sided P < 0.05 considered statistically significant.

Supplementary Material 3. Supplementary Table 1. Genomic profiling and variant characteristics detected in mesothelioma patients. Genetic profiling was conducted on mesothelioma FFPE tissues and effusion‑derived cell blocks using targeted sequencing panels of varying coverage. Somatic variants were categorized into Tier I–IV per AMP/ASCO/CAP consensus criteria, and germline variants were assessed in accordance with the 2015 ACMG/AMP guidelines. FFPE: Formalin-fixed, paraffin-embedded tumor tissue; CB: Cell blocks from pleural/ascitic effusions; NA: Primary Site Unknow; LP: Likely Pathogenic; VUS: Variant of Uncertain Significance; S: Somatic mutation; G: Germline mutation

## Data Availability

The original datasets are available from the corresponding author on reasonable request.

## Glossary

AREs: Asbestos-related enterprises
ATM: Ataxia telangiectasia mutated
BAP1: BRCA1 associated protein 1
*BRCA1*: Breast cancer gene 1
*BRCA2*: Breast cancer gene 2
*CDKN2A*: Cyclin dependent kinase inhibitor 2A
*EWSR1*: Ewing sarcoma breakpoint region 1
FANCI: Fanconi anemia complementation group I
HE staining: Hematoxylin and eosin staining
LOESS: Locally estimated scatterplot smoothing
NF2: Neurofibromin 2
OS: Overall survival
PALB2: Partner and localizer of BRCA2
PFS: Progression-free survival
TP53: Tumor protein P53
