# Supplementary material for: Distinct epidemiologic patterns of mesothelioma: evidence from a 16-year provincial cohort in China
Source: BMC Cancer. 2026 Jun 15;26:1012. doi: 10.1186/s12885-026-16357-9 (PMC13501794; doi:10.1186/s12885-026-16357-9)
Supplement: Supplementary file 1 [file 12885_2026_16357_MOESM1_ESM.pdf]

Supplementary Figure 1

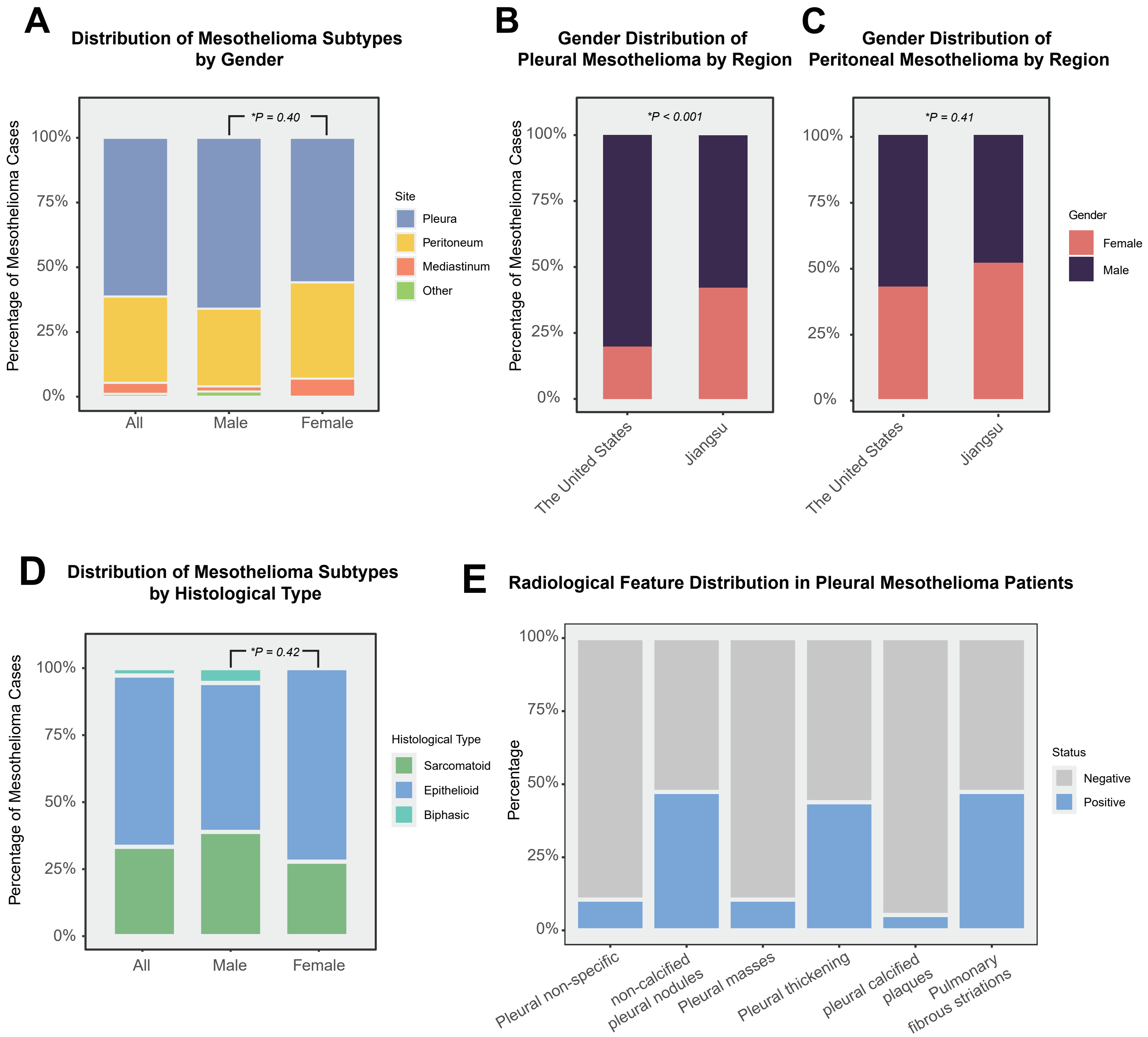

Supplementary Figure 1. Combined analysis of gender and mesothelioma subtypes. (A) The overall distribution of mesothelioma subtypes was similar between males and females, with no significant difference (χ^2 = 2.943, P = 0.40). **(B,C)** Regional variations in gender-specific proportions of pleural and peritoneal mesothelioma. A significant difference in gender distribution was observed between Jiangsu and the United States in the pleural mesothelioma group (χ^2 = 16.172, P < 0.001). **(D)** Distribution of histological subtypes of mesothelioma stratified by sex, showing no significant difference between male and female patients (χ^2 = 1.7246, P = 0.42). **(E)** Proportional distribution of radiological findings identified in pleural mesothelioma. “Pleural non-specific” is defined as the absence of identifiable pleural abnormalities or the presence of isolated pleural effusion at initial presentation. Differences were analyzed using the χ^2 test, with two-sided P < 0.05 considered statistically significant.
