# Supplementary material for: Distinct epidemiologic patterns of mesothelioma: evidence from a 16-year provincial cohort in China
Source: BMC Cancer. 2026 Jun 15;26:1012. doi: 10.1186/s12885-026-16357-9 (PMC13501794; doi:10.1186/s12885-026-16357-9)
Supplement: Supplementary file 2 [file 12885_2026_16357_MOESM2_ESM.pdf]

Supplementary Figure 2

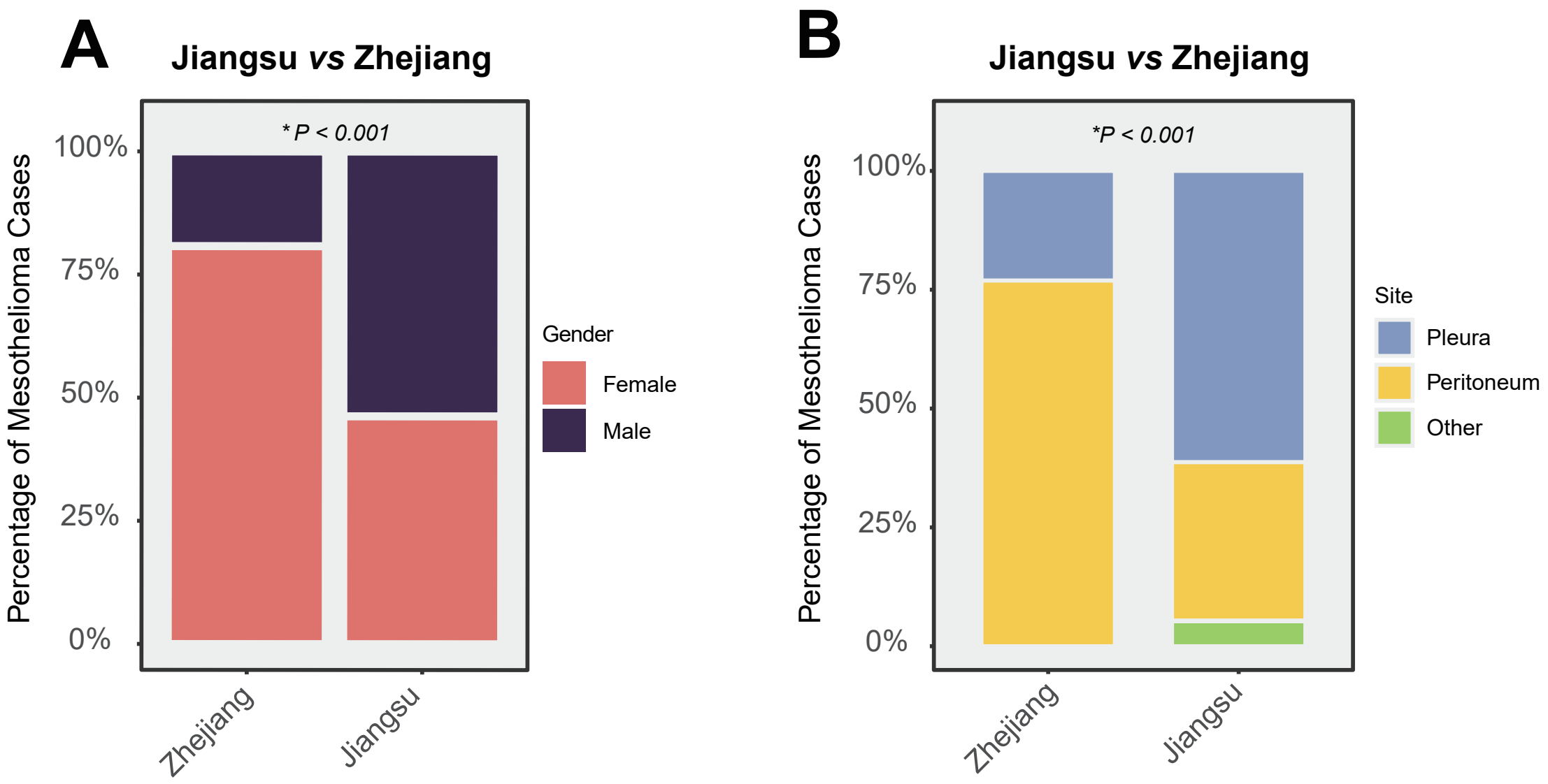

Supplementary Figure 2. **Comparison between Jiangsu and Zhejiang provinces in China. (A, B)** The gender and anatomical distribution patterns in mesothelioma were compared between Zhejiang and Jiangsu provinces in China, with both comparisons reaching statistical significance (Gender: χ^2 = 15.004, P<0.001; Anatomical distribution: χ^2 = 25.972, P<0.001). Differences were analyzed using the χ^2 test, with two-sided $P < 0.05$ considered statistically significant.
